# Efficacy of sodium-glucose cotransporter-2 inhibitors on functional mitral regurgitation associated with heart failure: a systematic review and meta-analysis of randomized controlled trials

**DOI:** 10.3389/fcvm.2026.1976876

**Published:** 2026-10-21

**Authors:** Charlie Kajo, Ahmed J. Alaraibi, Osama T. Almutairi, Yaqeen H. Alaraibi, Abdullah Alibrahim, Yousef Kh A. Almutawah, Salem W. S. Alasfar, Mustafa Wasif Ali, Aseel Hajjiri, Mohammad Alqaryuti, Abdullatif Alfehaid, Dana Arekat

**Affiliations:** School of Medicine, Royal College of Surgeons in Ireland, Medical University of Bahrain, Busaiteen, Bahrain

**Keywords:** dapagliflozin, empagliflozin, functional mitral regurgitation, heart failure, secondary mitral regurgitation, SGLT2 inhibitors, valvular outcomes

## Abstract

**Background:**

Functional mitral regurgitation (FMR) commonly complicates heart failure (HF) and portends a poor prognosis. While sodium-glucose cotransporter-2 inhibitors (SGLT2i) improve clinical outcomes and induce cardiac reverse remodeling in HF, their specific effect on FMR associated with HF severity remains uncertain.

**Methods:**

Five databases were searched from inception to 21 July 2026 for randomized controlled trials (RCTs) evaluating SGLT2i in adult patients with HF and chronic FMR on background guideline-directed medical therapy (GDMT). Primary outcomes were changes from baseline in effective regurgitant orifice area (EROA) and regurgitant volume (RV). Secondary clinical outcomes included HF hospitalization and cardiovascular (CVS) death. Random-effects models were used. Risk of bias was evaluated using the Cochrane RoB 2 tool, and certainty of evidence was assessed using GRADE.

**Results:**

Three RCTs involving 455patients were included. SGLT2i therapy was associated with a statistically significant reduction in EROA (MD −0.086 cm^2^, 95% CI −0.150 to −0.022) and RV (MD −10.87 mL, 95% CI −17.32 to −4.43), but substantial heterogeneity was observed (I^2^ = 72.5% and I^2^ = 63.5%, respectively). On leave-one-out sensitivity analyses, statistical significance for both primary valvular outcomes was lost after omitting the EFFORT trial. The certainty of evidence was rated as low.

**Conclusion:**

Addition of SGLT2i to GDMT was associated with reductions in FMR severity in pooled analyses; however, these findings were not robust in sensitivity analyses and are limited by low certainty of evidence. The apparent reduction in clinical events requires cautious interpretation due to event sparsity. These results should be considered hypothesis-generating rather than confirmatory, warranting further validation in large-scale, prospective trials.

**Systematic Review Registration:**

https://www.crd.york.ac.uk/PROSPERO/view/CRD420261462002, PROSPERO CRD420261385207

## 1 Introduction

Functional mitral regurgitation (FMR), also referred to as secondary mitral regurgitation, occurs in approximately one-third of patients with heart failure (HF) and contributes to disease progression and adverse clinical outcomes (1). FMR results from left ventricular (LV) remodeling, where an imbalance between leaflet tethering and LV closing forces reduces mitral leaflet coaptation (2, 3). Progressive LV remodelling leads to displacement of the papillary muscles, thereby, promoting leaflet tethering, mitral annular dilation and flattening together with reduced valvular closing forces ultimately resulting in failure of leaflet coaptation and the development of FMR (4). Increasing FMR severity is linked to a poor prognosis in patients with HF (1).

The current standard treatment for FMR is guideline-directed medical therapy (GDMT), which includes beta-blockers, mineralocorticoid receptor antagonists, renin-angiotensin system inhibitors, Sodium-glucose cotransporter-2 (SGLT2) inhibitors (SGLT2i), and, when indicated, cardiac resynchronization therapy (5). Although GDMT has achieved at least one grade reduction in severity in 39.3% of patients in the study by Spinka et al. (6). Despite receiving GDMT, some patients continue to experience symptomatic FMR, highlighting the need for additional therapeutic strategies (5).

Initially, SGLT2i were used to treat type 2 diabetes mellitus (T2DM) (7). However, they have been shown to reduce HF hospitalizations and cardiovascular mortality (7). In addition, evidence from a meta-analysis of randomized controlled trials (RCTs) suggests that SGLT2i are associated with reverse cardiac remodeling in patients with HF (8). The cardioprotective effects of SGLT2i have been linked to improvements in cardiac cell metabolism and reductions in cardiac fibrosis and cardiac cell necrosis (9).

Previous RCTs, including the DEFORM and EFFORT trials have suggested improvement of FMR following SGLT2i therapy (10, 11). However, evidence remains limited due to small sample sizes, varying follow-up durations, and limited assessment of clinical outcomes as trials were designed as mechanistic rather than outcome trials (10, 11). As a result, the overall efficacy and safety of SGLT2i for the management of FMR remain uncertain (10, 11). Therefore, we conducted a systematic review and meta-analysis to objectively assess and synthesize the available evidence on the role of SGLT2 inhibitors in patients with functional mitral regurgitation associated with heart failure.

## 2 Methods

### 2.1 Study registration

The protocol for this systematic review and meta-analysis was prospectively registered in PROSPERO (CRD420261385207). It was conducted in accordance with the Preferred Reporting Items for Systematic Reviews and Meta-Analyses 2020 statement and the recommendations of the Cochrane Handbook for Systematic Reviews of Interventions (12, 13).

### 2.2 Literature search and study selection

A comprehensive literature search was conducted by CKA in five different electronic databases: PubMed, Embase, Scopus, Web of Science, and the Cochrane Central Register of Controlled Trials from database inception to 21 July, 2026. The search strategy combined controlled vocabulary terms including Medical Subject Headings (MeSH) terms and free-text keywords related to heart failure, sodium-glucose cotransporter-2 inhibitors, functional or secondary mitral regurgitation. Relevant drug-specific terms, including dapagliflozin, empagliflozin, ertugliflozin, canagliflozin, gliflozin, and SGLT2 inhibitor, were also incorporated. No date or lanuage restrictions were applied. The search string was adapted to of each database. The complete database-specific search strategies and the number of records retrieved are provided in Supplementary Table S1.

All identified records were imported into Covidence for duplicate removal and screening (14). Duplicate records were removed automatically within Covidence and through additional manual review. Two reviewers independently screened the titles and abstracts of all remaining records against the predefined eligibility criteria. Reports considered potentially eligible underwent independent full-text assessment. Reasons for exclusion at the full-text stage were recorded. Any disagreement during title and abstract screening or full-text assessment was resolved through consultation of the senior author. The study selection process was documented using the PRISMA 2020 flow diagram (Figure 1).

**Figure 1:**
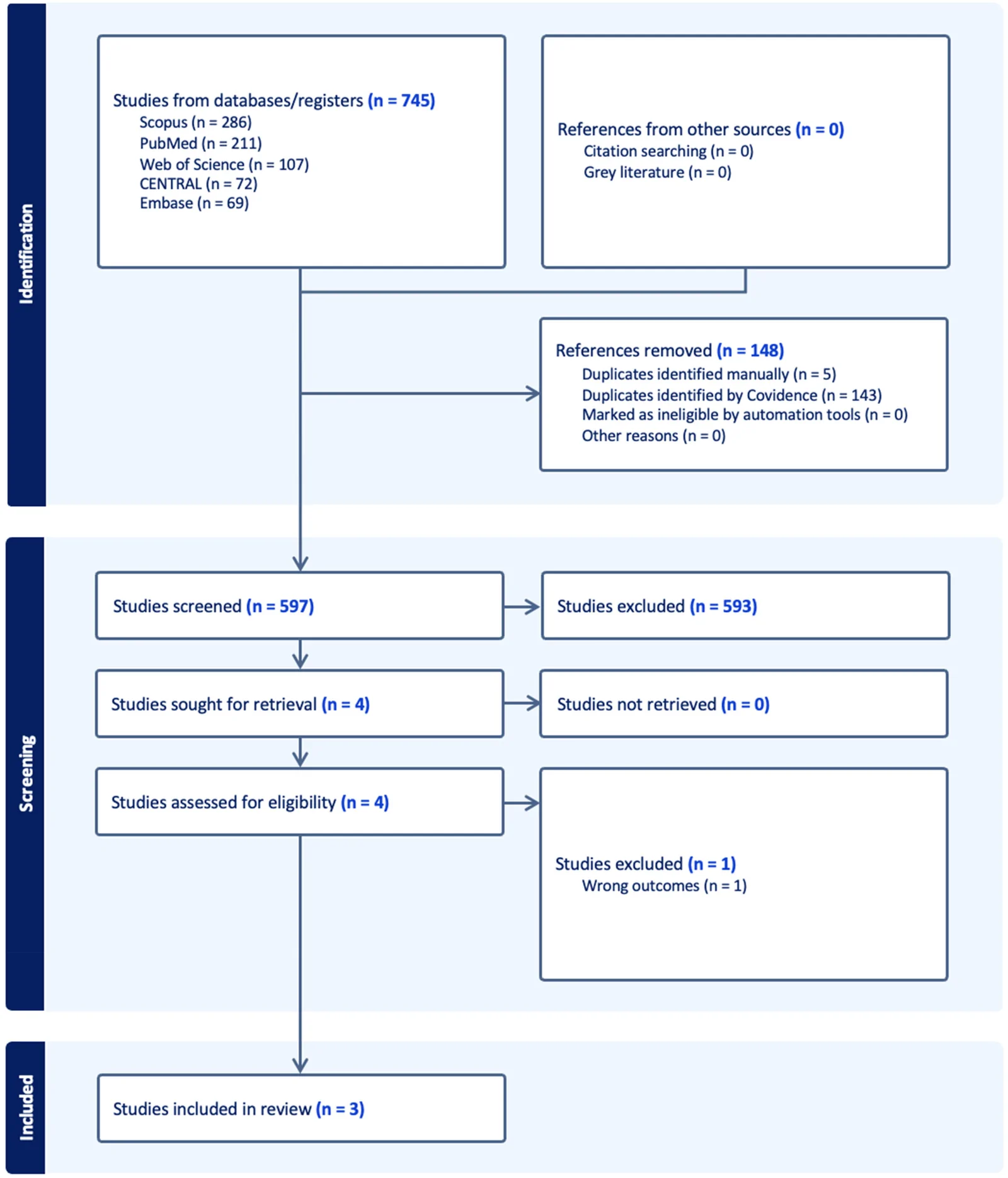
PRISMA 2020 flow diagram of study selection.

### 2.3 Eligibility criteria

Based on the PICO framework, studies were included if they satisfied the following requirements. The population (P) comprised of adult patients diagnosed with HF of any type and concomitant chronic FMR confirmed by echocardiography. FMR was defined as regurgitation arising from ventricular or atrial remodeling in the presence of structurally normal mitral valve leaflets and chordal apparatus. The intervention (I) included any approved orally administered SGLT2i, including dapagliflozin, empagliflozin, ertugliflozin, or canagliflozin, administered in addition to background GDMT, while the comparator (C) was a matching placebo plus GDMT or GDMT alone. The primary outcomes (O) were changes from baseline in effective regurgitant orifice area (EROA), measured in cm^2^, and mitral regurgitant volume (RV), measured in mL, while the secondary clinical outcomes included the incidence of hospitalization due to HF and cardiovascular (CVS) death. Only RCTs were included.

Studies were excluded if they were non-randomized, observational, retrospective, or presented as reviews, case reports, conference abstracts without sufficient extractable data, animal studies, or *in vitro* investigations. Studies enrolling exclusively pediatric participants were excluded. Trials involving primary or organic mitral valve disease, including rheumatic valve disease, mitral valve prolapse, severe annular calcification, or other structural leaflet abnormalities, were not eligible.

### 2.4 Data extraction

Four reviewers independently extracted data from each included study using a standardized Microsoft Excel sheets. Any discrepancies between reviewers were resolved through discussion and re-examination of the literature with referral to a senior reviewer when necessary. Three extraction sheets were formulated to collect outcomes data, study characteristics, and patient baseline characteristics.

Variables in the study characteristics included country, year of publication, sample sizes (both arms), intervention and control details, follow-up duration, and key inclusion and exclusion criteria for each included RCT. Patient baseline characteristics included patients demographical information, clinical characteristics and comorbidities, baseline biochemical markers, baseline echocardiographic parameters, and baseline medications. Finally, outcomes data extracted for continous endpoints included means and standard deviations (SD) of change from baseline, number of patients in each arm, mean differences (when reported), and 95% confidence intervals (CI) of effect estimates. Moreover, standard errors were calculated using algebraic conversion from reported 95% CI or derived using the standard formula for the variance of the difference between two independent means ($SE=\sqrt{SD_{1}^{2}/n_{1}+SD_{2}^{2}/n_{2}}$). For dichotomous endpoints, event counts and total number of patients for each arm were extracted.

### 2.5 Bias and certainty assessments

Two reviewers independently assessed the risk of bias in each included RCT using the Cochrane Risk of Bias 2 (RoB2) tool (15). Five domains were evaluated: bias arising from the randomization process, bias due to deviations from the intended interventions, bias due to missing outcome data, bias in measurement of the outcome, and bias in selection of the reported result. Each domain and the overall risk of bias were judged as low risk, some concerns, or high risk of bias. Disagreements were resolved through consultation with a third senior reviewer. Results were summarized using a traffic-light plot and a weighted domain-level graph that were generated using the RobVis web tool (16).

The certainty of evidence for each pooled outcome was evaluated using the Grading of Recommendations Assessment, Development and Evaluation (GRADE) approach (17). As the evidence originated from RCTs, certainty was initially considered high and was downgraded when appropriate for limitations related to risk of bias, inconsistency, indirectness, imprecision, or publication bias. The final certainty of evidence was classified as high, moderate, low, or very low.

### 2.6 Data synthesis

Meta-analyses were performed using R software within RStudio, version 2025.09.2 + 418, with the meta and metafor packages (18, 19). Quantitative syntheses for each outcome were undertaken when at least two clinically and methodologically comparable studies reported it. Effect estimates were presented with 95% CI, and forest plots were generated for all pooled analyses.

For continuous outcomes reported in a common unit, including change in EROA and RV, pooled treatment effects were expressed as mean differences (MD) with 95% CI. Study-level MDs and their corresponding standard errors were utizlised to coduct the analyses using generic inverse-variance weighting. For dichotomous outcomes, including HF hospitalization and CVS death, pooled effects were expressed as risk ratios (RR) with 95% CI. Event counts and treatment-group denominators were utilized to conduct both analyses using the Mantel-Haenszel method.

Random-effects models were used for all meta-analyses to account for anticipated clinical and methodological variability across trials. Statistical heterogeneity was evaluated using Cochran's *Q* test and quantified using the I^2^ and *τ*^2^ tools. I^2^ values greater than 50% were considered indicative of substantial between-study heterogeneity. However, it was interpreted alongside differences in participant characteristics, intervention type, follow-up duration, and baseline mitral regurgitation severity. The robustness of the effect estimates of our primary endpoints was evaluated using leave-one-out (LOO) sensitivity analyses, in which each included study was sequentially removed and the meta-analysis was recalculated. Publication bias was not quantitatively assessed, as the number of studies for each outcome was fewer than 10 (20).

## 3 Results

### 3.1 Study selection

The database search identified 745 records, including 286 from Scopus, 211 from PubMed, 107 from Web of Science, 72 from CENTRAL, and 69 from Embase. No additional records were identified through citation searching or grey-literature sources. After removal of 148 duplicates, of which 143 were identified using Covidence and five were removed manually, 597 records underwent title and abstract screening. A total of 593 records were excluded at this stage.

Four reports were subsequently sought for retrieval, and all were successfully obtained and assessed for eligibility. One report was excluded at the full-text stage because it did not report the outcomes required for inclusion. Three RCTs were therefore included in the systematic review and quantitative meta-analysis (10, 11, 21). The complete selection process is presented in the PRISMA flow diagram in Figure 1.

### 3.2 Study characteristics

Our included studies had initially randomized a total of 472 participants, of whom 455 completed follow-up and were therefore included in the quantitative analysis. The trials were conducted across 3 different countries, Egypt, South Korea and China. The research period spans from 2024 to 2025. The included RCTs varied in design: one was a multicenter, double-blind trial (EFFORT Trial), while two were open-label trials with blinded outcome assessment, conducted across two centers (DEFORM trial) and a single center (Abou Warda et al.) respectively. The main design and baseline characteristics of the included trials are summarized in Table 1.

**Table 1:** Summary of the included studies.

Study		EFFORT Trial	DEFORM trial	Abou Warda et al.
Country		South Korea	China	Egypt
Year of Publication		2024	2025	2025
Design		Prospective, six-center, double blinded, placebo-controlled trial	Prospective, double-center, open-label, parallel-controlled trial	Prospective, single-center, open-label, parallel-controlled trial
Sample Size	SGLT2i Arm	63	52	160
	Control Arm	65	52	80
	Sum	128	104	240
Intervention Details		Ertugliflozin + GDMT	Dapagliflozin, 10 mg, OD + GDMT	Dapagliflozin, 10 mg, OD + GDMT
Control Details		Placebo + GDMT	GDMT alone	GDMT alone
Follow-up Duration		12 months	3 months	6 months
Primary Outcome/s		Change in EROA	Change in EROA	The difference in the indexed LV mass among both arms
Key Inclusion Criteria		• Age ≥ 20 • Stable HF (NYHA class II–III) • EF = (35% to <50%) • Functional MR with EROA>0.1cm^2^ for > 6 months • Receiving GDMT for ≥ 4 weeks before enrollment	• Age > 18 • EROA of MR ≥ 0.2 cm^2^ • LVEF < 60% • Normal structure of the mitral valve leaflets + chordae tendineae • Receiving GDMT for FMR + a stable optimized dose of *β*-blockers, RAS inhibitors, and MRAs ≥ 2 weeks • No use of intravenous anti-HF(past 2 weeks)	• Chronic symptomatic HF (NYHA Functional Class II-III) + LV dysfunction •HFrEF or HFmrEF • Right ventricular impairment and functional mitral regurgitation • Receiving GDMT for ≥ 3 months • NT-proBNP level ≥ 600 pg/mL, for those with atrial fibrillation or flutter, threshold was higher at 900 pg/mL
Exclusion Criteria		• SBP < 100mmHg • LVEF < 35% • Glycated Hemoglobin > 10.5% • eGFR <45 mL/min/1.73 m^2^ • Structural mitral valve disease • Previous valve intervention • History of ketoacidosis • Previous hospitalization within 6 weeks • NYHA IV symptoms • History of heart related disease • Plan for a cardiovascular intervention	• Allergy to dapagliflozin • History of angioedema • Current use of dapagliflozin or other SGLT2 inhibitors • Mitral valve damege • CKD with eGFR <30 mL/min/1.73 m2 • Acute myocardial infarction or acute myocarditis (past 3 months) • Recent or planned revascularization procedure • Concomitant significant aortic valve disease • Untreated hyperthyroidism • Pregnancy or lactation	• Hospitalization for HF within the recent three months • Acute decompensated cardiac failure • Cardiogenic shock • Recent coronary interventions • Valvular surgeries • Stroke • Ablation procedures • Transient ischemic attack • Presence of devices (such as pacemakers) within the past six months •HFpEF • eGFR < 30 mL/min/1.73 m^2^ • End stage kidney faliure or liver faliure • Hypersensitivity to SGLT2 inhibitors • Pregnancy or lactation

CKD, chronic kidney disease; EF, ejection fraction; eGFR, estimated glomerular filtration rate; EROA, effective regurgitant orifice area; FMR, functional mitral regurgitation; GDMT, guideline-directed medical therapy; HF, heart failure; HFmrEF, heart failure with mildly reduced ejection fraction; HFpEF, heart failure with preserved ejection fraction; HFrEF, heart failure with reduced ejection fraction; LV, left ventricular; LVEF, left ventricular ejection fraction; MR, mitral regurgitation; MRA, mineralocorticoid receptor antagonist; NT-proBNP, N-terminal pro-B-type natriuretic peptide; NYHA, New York Heart Association; OD, once daily; RAS, renin-angiotensin system; SBP, systolic blood pressure; SGLT2i, sodium-glucose cotransporter-2 inhibitor.

Two RCTs utilized Dapagliflozin, while one used Ertugliflozin. In the improvement of EROA, all treatments were compared to the use of GDMT, while one study also implemented a placebo in the control group (EFFORT Trial).

The eligibility criteria differed slightly, but all trials enrolled patients with FMR and chronic HF on stable GDMT. The EFFORT Trial included patients with FMR of >6 months duration and mildly reduced LV ejection fraction, while the DEFORM trial was designed to study patients with an EROA ≥0.20 cm^2^ and LV ejection fraction <60%. The Abou Warda et al. study enrolled patients with HF with symptoms, reduced or mildly reduced ejection fraction, and right ventricular dysfunction. Frequent exclusion criteria were pregnancy, severe renal impairment, recent cardiovascular events or procedures, structural mitral valve disease, and intolerance to SGLT2i.

### 3.3 Patients baseline characteristics

The baseline demographic characteristics were balanced between the intervention and control groups. The pooled mean age of patients was 61.5 years, with the majority being males (70.3%). Mean body mass index was consistently within the overweight range (pooled mean 25.3 kg/m^2^) without clinically important differences between treatment arms.

The burden of cardiovascular comorbidities was comparable across studies; within the pooled cohort, hypertension was present in 41.7%, diabetes mellitus in 29.9%, atrial fibrillation in 26.3%, and coronary artery disease in 47.4% where reported. Baseline systolic and diastolic blood pressures were consistent between study groups and were indicative of stable chronic heart failure populations receiving optimized medical therapy.

Baseline laboratory values, namely mean hemoglobin and HbA1c, were similarly balanced between groups where reported. Echocardiographic parameters confirmed the presence of chronic functional mitral regurgitation in all participants before randomization, though regurgitation severity differed between studies due to variations in inclusion criteria. A conclusive summary of the baseline demographic, clinical, and statistical characteristics is provided in Table 2.

**Table 2:** Baseline characteristics of the included studies.

Study		EFFORT Trial		DEFORM trial		Abou Warda et al.	
Arm		Interventional	Control	Interventional	Control	Interventional	Control
Demographic Information							
Age (years), Mean (SD)		65.4 ± 11.5	67.3 ± 11.0	61.98 ± 13.71	65.31 ± 13.87	58.0 ± 11.2	58.0 ± 9.8
Gender	Male, *n* (%)	40 (63.5%)	38 (58.5%)	45 (86.5%)	38 (73.1%)	112 (70.0%)	59 (73.7%)
	Female, *n* (%)	23 (36.5%)	27 (41.5%)	7 (13.5%)	14 (26.9%)	48 (30.0%)	21 (26.3%)
BMI (kg/m2), Mean (SD)		24.3 ± 2.8	25.0 ± 3.3	23.74 ± 2.44	24.21 ± 2.42	25.7 ± 3.6	27.2 ± 5.3
Clinical Characteristics & Comorbidities							
Heart Rate (beats/min), Mean (SD)		75.3 ± 12.1	73.8 ± 13.8	NR	NR	80.0 ± 16.2	80.0 ± 12.5
SBP (mmHg), Mean (SD)		118.7 ± 13.9	124.0 ± 13.0	111.83 ± 13.25	107.46 ± 11.78	117.0 ± 12.3	110.0 ± 15.1
DBP (mmHg) Mean (SD)		69.3 ± 11.0	70.9 ± 9.3	58.69 ± 10.69	53.31 ± 12.56	75.0 ± 8.2	75.0 ± 12.2
Hypertension, *n* (%)		24 (38.1%)	33 (51.6%)	21 (41.2%)	19 (36.5%)	73 (45.6%)	27 (33.7%)
Diabetes, *n* (%)		7 (11.1%)	9 (13.8%)	20 (38.5%)	17 (32.7%)	65 (40.6%)	23 (28.7%)
Coronary Artery Disease, *n* (%)		24 (38.1%)	29 (44.6%)	28 (53.8%)	29 (55.8%)	NR	NR
Atrial Fibrillation, *n* (%)		33 (52.4%)	32 (49.2%)	14 (26.9%)	19 (36.5%)	21 (13.1%)	5 (6.2%)
Baseline Biochemical Markers							
Haemoglobin (g/dL), Mean (SD)		13.5 ± 1.6	13.6 ± 1.6	NR	NR	12.6 ± 1.75	12.8 ± 1.75
Haematocrit (%), Mean (SD)		NR	NR	NR	NR	37.1 ± 5.2	37.3 ± 5.3
HbA1c (%), Mean (SD)		NR	NR	6.48 ± 1.21	6.29 ± 1.10	6.3 ± 1.6	6.3 ± 2.2
eGFR (mL/min/1.73 m2), Mean (SD)		NR	NR	64.47 ± 22.05	67.50 ± 27.54	72.5 ± 28.6	74.5 ± 16.6
LDL-C (mg/dL), Mean (SD)		85.9 ± 28.8	87.0 ± 32.0	94.7 ± 34.4	100.5 ± 34.8	NR	NR
NT-proBNP (pg/m), Mean (SD)		830 ± 1057	1126 ± 1725	3872.6 ± 3718.6	3068.0 ± 3214.1	6033.2 ± 1017.6	5916.6 ± 909.3
Serum Creatinine (mg/dL), Mean (SD)		0.92 ± 0.20	0.96 ± 0.19	1.24 ± 0.39	1.26 ± 0.70	1.10 ± 0.34	1.11 ± 0.23
Baseline Echocardiographic Parameters							
EROA (cm2), Mean (SD)		0.20 ± 0.10	0.20 ± 0.12	0.296 ± 0.085	0.291 ± 0.075	0.43 ± 0.13	0.45 ± 0.23
RV (mL), Mean (SD)		33.7 ± 17.0	36.9 ± 23.1	43.6 ± 13.8	44.2 ± 12.9	52.0 ± 13.5	50.0 ± 17.7
LVEF (%), Mean (SD)		42.4 ± 7.5	41.9 ± 8.3	36.6 ± 11.8	39.0 ± 13.4	33.3 ± 7.8	32.5 ± 6.8
GLS (%), Mean (SD)		−12.0 ± 2.9	−12.9 ± 3.7	NR	NR	−11.04 ± 2.7	−10.7 ± 2.5
LVESV (mL), Mean (SD)		54.2 ± 22.9	56.4 ± 29.9	129.8 ± 59.2	113.1 ± 50.7	141.0 ± 52.2	145.1 ± 50.8
LVEDV (mL), Mean (SD)		89.9 ± 29.2	93.7 ± 37.5	204.8 ± 62.2	185.9 ± 63.6	207.0 ± 66.6	198.0 ± 82.1
Baseline Medications							
ACEI/ARB/ARNI, *n* (%)		ACEI:3 (4.8%) ARB:25 (39.7%) ARNI:27 (42.9%)	ACEI:4 (6.2%) ARB:24 (36.9%) ARNI:28 (43.1%)	ACEI/ARB:2 (3.8%) ARNI:45 (86.5%)	ACEI/ARB:3 (5.8%) ARNI:42 (80.8%)	136 (85.0%)	70 (93.3%)
Beta Blocker, *n* (%)		53 (84.1%)	54 (83.1%)	48 (92.3%)	46 (88.5%)	129 (80.6%)	58 (77.3%)
Statin, *n* (%)		NR	NR	35 (67.3%)	34 (65.4%)	NR	NR
Diuretic, *n* (%)		45 (71.4%)	54 (83.1%)	44 (84.6%)	41 (78.8%)	131 (81.8%)	68 (90.6%)
MRA, *n* (%)		33 (52.4%)	35 (53.8%)	46 (88.5%)	43 (82.7%)	113 (70.6%)	56 (74.6%)

ACEI, angiotensin-converting enzyme inhibitor; ARB, angiotensin receptor blocker; ARNI, angiotensin receptor-neprilysin inhibitor; BMI, body mass index; CAD, coronary artery disease; DBP, diastolic blood pressure; eGFR, estimated glomerular filtration rate; EROA, effective regurgitant orifice area; GLS, global longitudinal strain; HbA1c, glycated hemoglobin; LDL-C, low-density lipoprotein cholesterol; LVEF, left ventricular ejection fraction; LVEDV, left ventricular end-diastolic volume; LVESV, left ventricular end-systolic volume; MRA, mineralocorticoid receptor antagonist; NR, not reported; NT-proBNP, N-terminal pro-B-type natriuretic peptide; RV, regurgitant volume; SBP, systolic blood pressure; SD, standard deviation.

### 3.4 Primary outcomes

Three RCTs were included in the change in EROA analysis which included a total of 455 patients (SGLT2i = 269, control = 186) (Figure 2A). Using random-effects model, SGLT2i significantly reduced EROA compared to the control group (MD −0.086 cm^2^, 95% CI −0.15 to −0.022, *p* = 0.0089). However, substantial heterogeneity was observed after pooling the effect estimates (I^2^ = 72.5%). The LOO analysis confimed the overall direction of effect favoring SGLT2i across all iterations (MD = −0.060 to −0.118 cm^2^) and identified Abou Warda et al. as the main driver for between-study heterogenity (Figure 3A). Omitting this trial significantly reduced the heterogeneity from I^2^ = 72.5% to 46.9% (*τ*^2^ = 0.0003). In addition, statistical significance was lost after the omission of the EFFORT trial (MD −0.103 cm^2^, 95% CI −0.236 to +0.029, *p* = 0.1251), with the confidence interval crossing the null value in alignment with the non-significant *p*-value.

**Figure 2:**
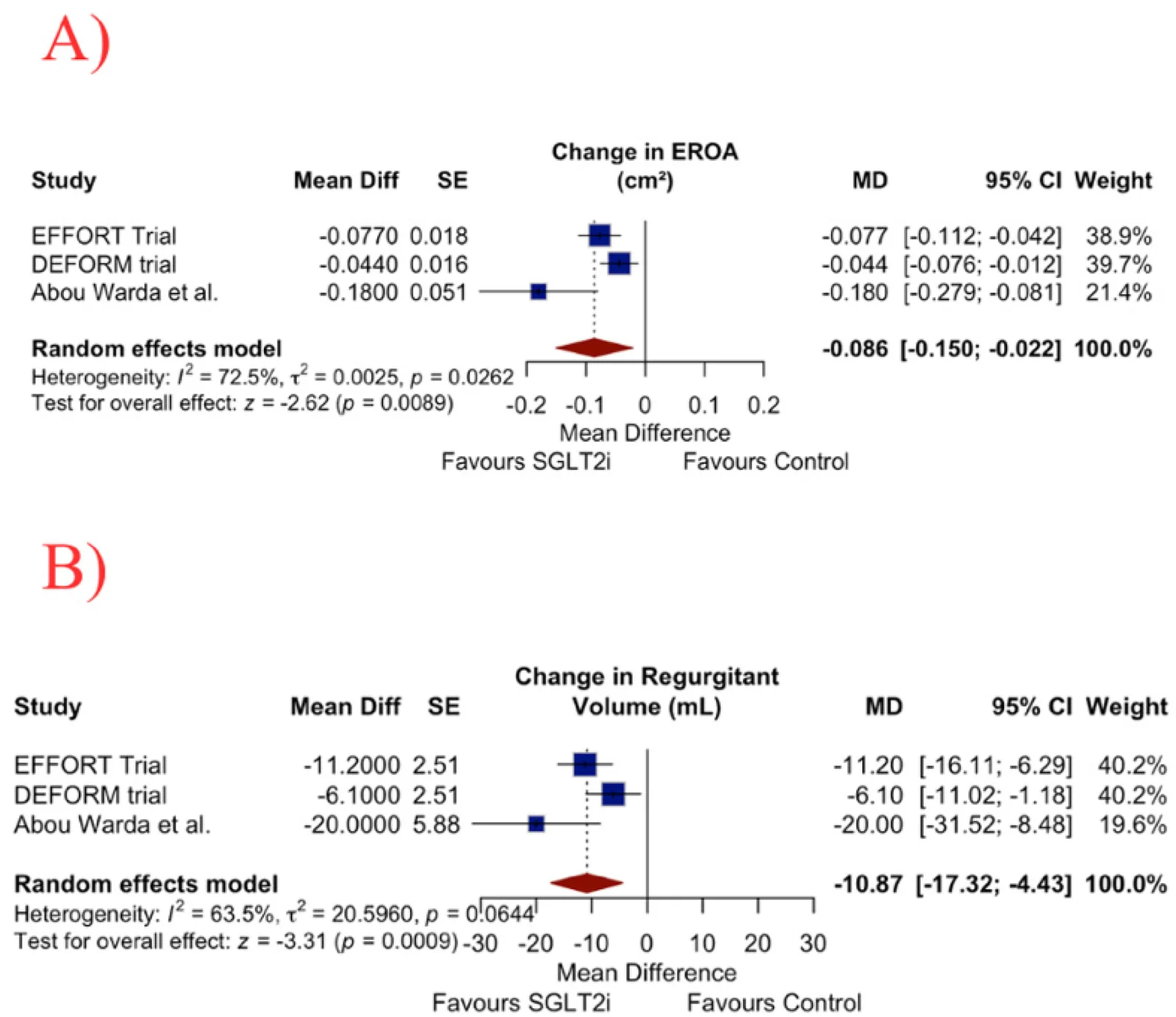
Forest plots of primary valvular outcomes comparing SGLT2 inhibitors with control in heart failure with functional mitral regurgitation. **(A)** Change in effective regurgitant orifice area (EROA, cm^2^), presented as mean difference (MD). **(B)** Change in regurgitant volume (RV, mL), presented as mean difference (MD). Squares represent individual study estimates, with sizes proportional to study weight, and horizontal lines indicate 95% confidence intervals (CI). Diamonds represent pooled estimates using a random-effects model. Values <0 favor SGLT2 inhibitors.

**Figure 3:**
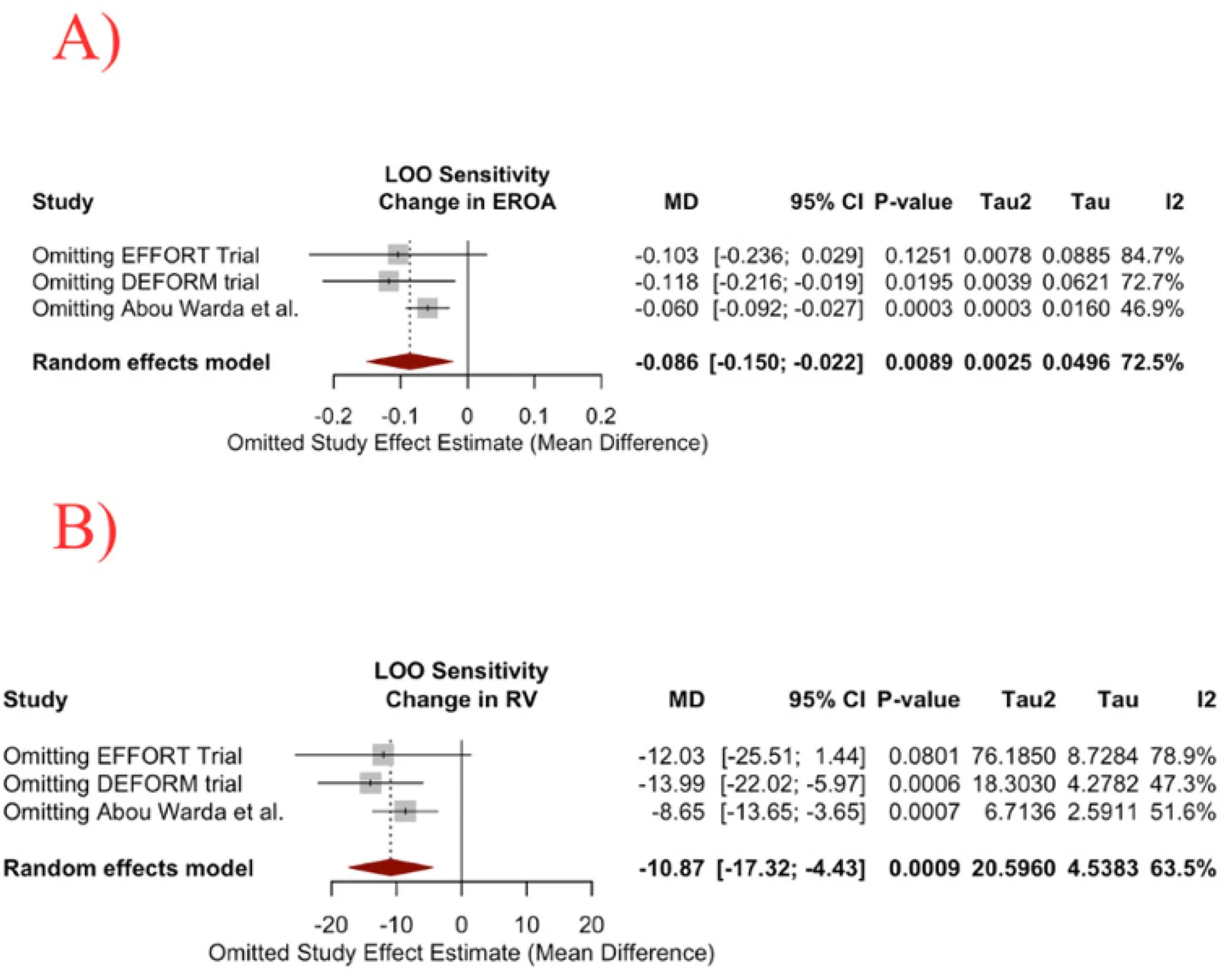
Leave-one-out (LOO) sensitivity analyses for primary valvular outcomes. **(A)** LOO analysis for change in effective regurgitant orifice area (EROA, cm^2^). **(B)** LOO analysis for change in regurgitant volume (RV, mL). Each row displays the recalculated pooled mean difference (MD), 95% confidence intervals (CI), *p*-value, and heterogeneity parameters (*τ*^2^, *τ*, I^2^) after omitting the specified study. Grey squares represent individual recalculations, and horizontal lines denote 95% CIs. The bottom red diamond represents the baseline pooled effect size across all included trials. Omitting the EFFORT Trial leads to a loss of statistical significance for both outcomes, highlighting its influence on the overall summary estimate.

All three trials contributed to the change in RV analysis (Figure 2B). SGLT2i also significantly reduced RV volume compared to the control group (MD −10.87 mL, 95% CI −17.32 to −4.43, *p* = 0.0009). Moderate to substantial heterogeneity was also observed in this analysis (I^2^ = 63.5%). In the LOO sensivity analysis, the overall direction of the effect estimate remained consistent favouring SGLT2i (Figure 3B). Omitting the EFFORT trial resulted in overall effect estimate of MD = −12.03 mL, however, statistical significance was lost (*p* = 0.0801).

### 3.5 Secondary outcomes

Across three RCTs, 455 patients were included for both secondary clinical outcomes (SGLT2i = 269, control = 186) (Figure 4). SGLT2i therapy was associated with a statistically significant 55% reduction in hospitalization due to HF when compared to the control group (RR 0.45, 95% CI 0.28 to 0.73, *p* = 0.0012) and no observed between-study heterogeneity (I^2^ = 0%) (Figure 4A). In addition, SGLT2i therapy significantly reduced the risk of CVS death by 74% relative to control (RR 0.26, 95% CI 0.12 to 0.58, *p* = 0.0009) with no observed heterogeneity (I^2^ = 0%) (Figure 4B).

**Figure 4:**
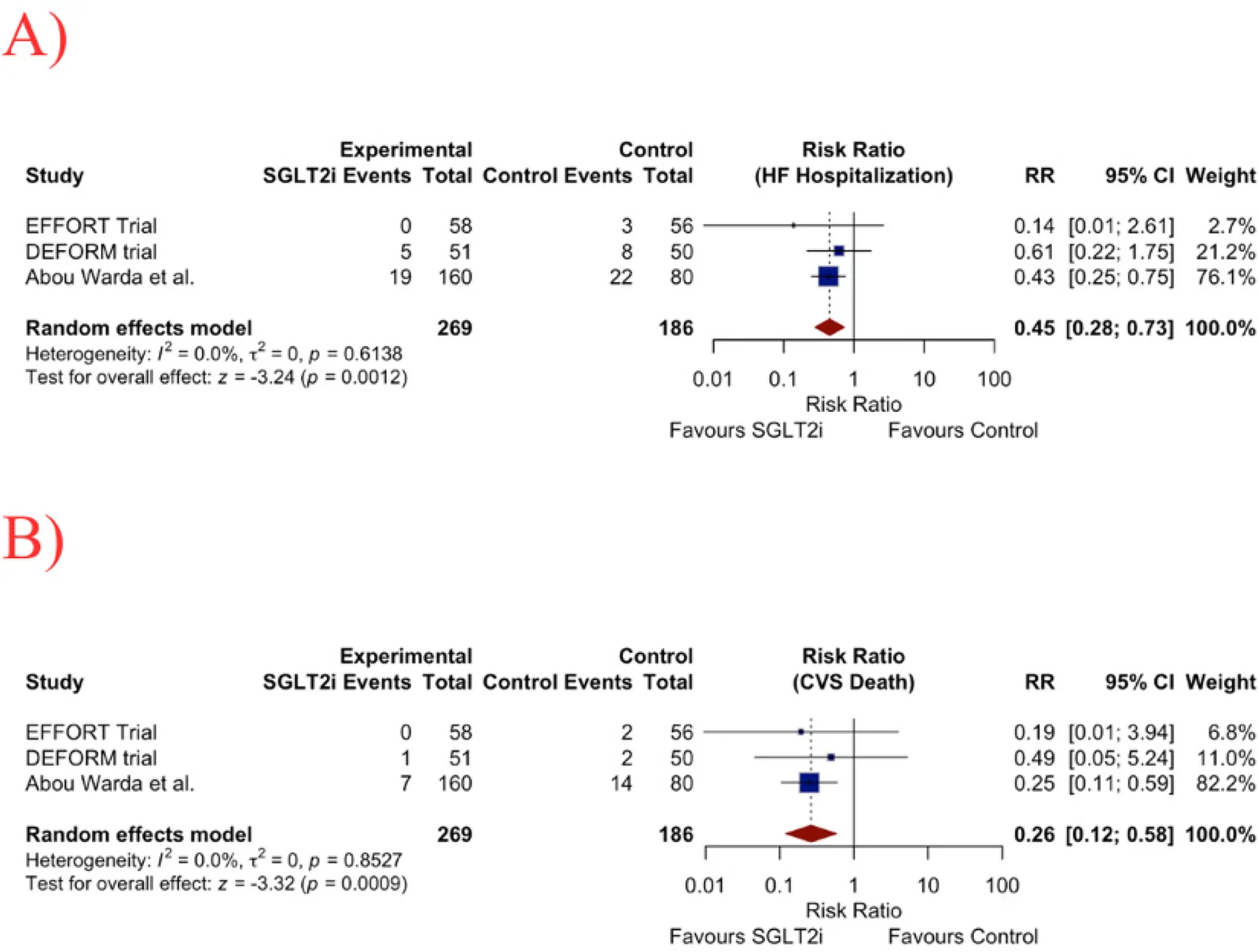
Forest plots of secondary clinical outcomes comparing SGLT2 inhibitors with control. **(A)** Meta-analysis for heart failure (HF) hospitalization, presented as risk ratio (RR). **(B)** Meta-analysis for cardiovascular (CVS) death, presented as risk ratio (RR). Squares represent individual study estimates, with sizes proportional to study weight, and horizontal lines indicate 95% confidence intervals (CI). Diamonds represent pooled estimates calculated using a Mantel-Haenszel random-effects model. Values <1 favor SGLT2 inhibitors.

### 3.6 Quality assessment and certainty of evidence

The overall risk of bias was low in two of the three included RCTs. The study by Abou Warda et al. was judged to raise some concerns overall because of concerns relating to missing outcome data and selection of the reported result. All three trials were assessed as having a low risk of bias in the randomization process, deviations from intended interventions, and measurement of outcomes. No trial was judged to have a high risk of bias in any domain or overall (Figure 5).

**Figure 5:**
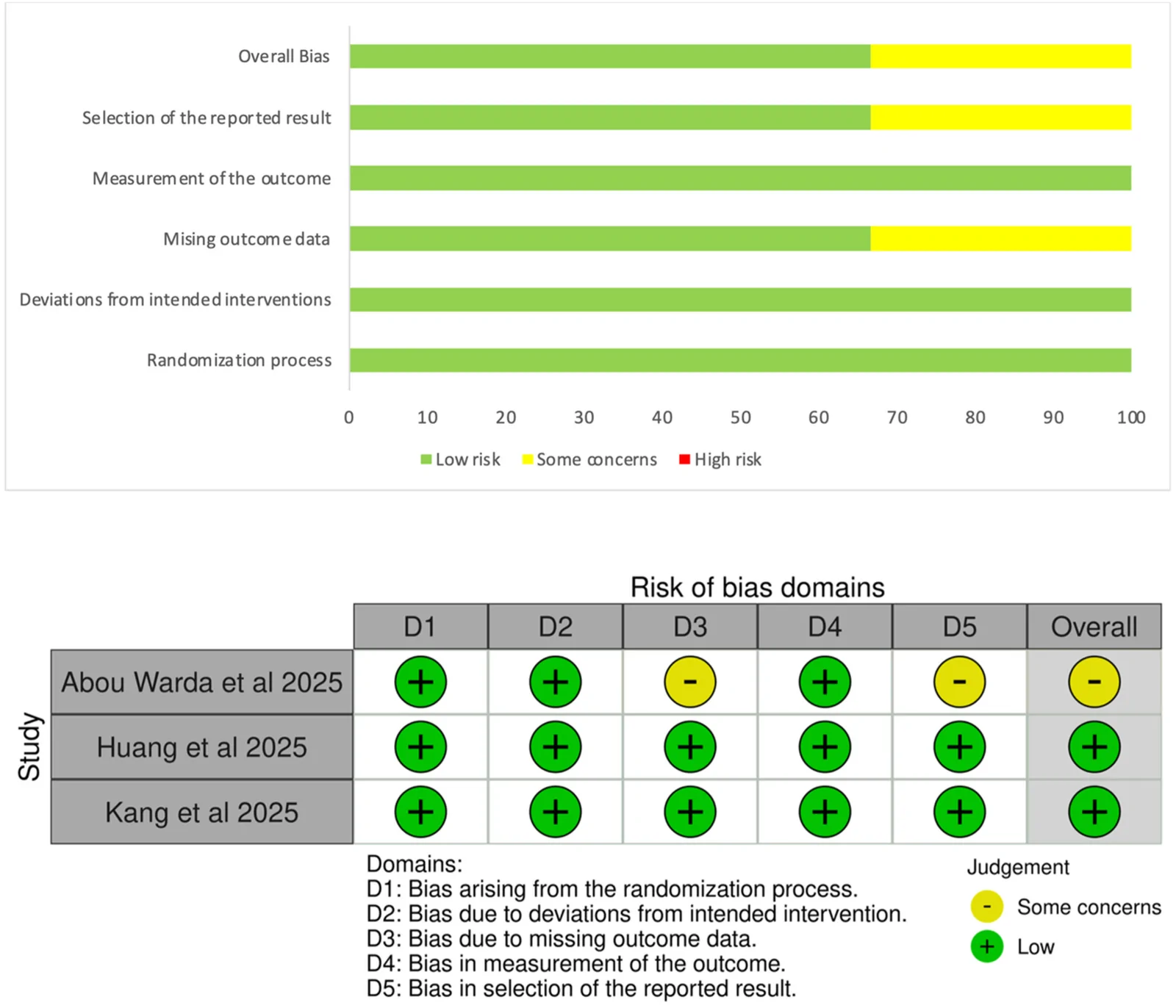
Risk of bias assessment for the included randomized controlled trials evaluated using the cochrane risk of bias 2 (roB 2) tool. (Top) Risk of bias graph presenting the aggregate proportion of studies categorized as having a low risk, some concerns, or a high risk of bias across each of the five evaluated domains and for overall bias. (Bottom) Risk of bias summary (traffic-light plot) detailing the specific methodological judgments for each included study across individual domains (D1-D5) and overall. Green circles (+) indicate a low risk of bias, and yellow circles (-) indicate some concerns.

The certainty of evidence for both primary valvular outcomes was rated as low. Although the evidence was derived from RCTs, it was downgraded for inconsistency because of substantial between-study heterogeneity and for imprecision because only three trials were available and the statistical significance of both pooled estimates was sensitive to exclusion of the EFFORT Trial. The certainty assessments for all the outcomes are summarized in Table 3.

**Table 3:** GRADE assessment for study endpoints.

Outcome (№ participants; № studies)	Study design	Risk of bias	Inconsistency	Indirectness	Imprecision	Publication bias	Effect estimate (95% CI)	Certainty of evidence
Primary outcomes								
Change in effective regurgitant orifice area (cm^2^) (455; 3 RCTs)	Randomized trials	Not serious ᵃ	Serious ᵇ	Not serious ᶜ	Serious ᵈ	Undetected ⁱ	MD −0.086 cm^2^ (95% CI −0.150 to −0.022) ʲ	⊕⊕◯◯ LOW
Change in regurgitant volume (mL) (455; 3 RCTs)	Randomized trials	Not serious ᵃ	Serious ᵇ	Not serious ᶜ	Serious ᵈ	Undetected ⁱ	MD −10.87 mL (95% CI −17.32 to −4.43) ʲ	⊕⊕◯◯ LOW
Secondary outcomes								
Heart failure hospitalization (455; 3 RCTs)	Randomized trials	Serious ᶠ	Not serious ᵍ	Not serious ᵉ	Serious ʰ	Undetected ⁱ	RR 0.45 (95% CI 0.28 to 0.73) ᵏ 177 per 1000 → 80 per 1000 98 fewer per 1000 (128 fewer to 48 fewer)	⊕⊕◯◯ LOW
Cardiovascular mortality (455; 3 RCTs)	Randomized trials	Serious ᶠ	Not serious ᵍ	Not serious ᵉ	Very serious ʰ	Undetected ⁱ	RR 0.26 (95% CI 0.12 to 0.58) ᵏ 97 per 1000 → 25 per 1000 72 fewer per 1000 (85 fewer to 41 fewer)	⊕◯◯◯ VERY LOW

CI, confidence interval; MD, mean difference; RCT, randomized controlled trial; RR, risk ratio. Certainty symbols: ⊕⊕◯◯ low; ⊕◯◯◯ very low. Anticipated absolute effects for dichotomous outcomes are calculated from the observed control-group risk (33/186 for heart failure hospitalization; 18/186 for cardiovascular mortality) and the pooled risk ratio.

## 4 Discussion

### 4.1 Summary of findings

This meta-analysis of three randomized trials enrolling 455 participants found that adding an SGLT2 inhibitor to guideline-directed medical therapy reduced effective regurgitant orifice area by 0.086 cm^2^ (95% CI −0.150 to −0.022) and regurgitant volume by 10.87 mL (95% CI −17.32 to −4.43). Both estimates carried substantial heterogeneity and the certainty of evidence was low. Heart failure hospitalization and cardiovascular death were less frequent with SGLT2 inhibition, although these analyses rested on few events and were dominated by a single trial. To our knowledge, this is the first quantitative synthesis of valvular endpoints with SGLT2 inhibitors; previous meta-analyses of this class have addressed ventricular remodeling (22), arrhythmia (23) and clinical outcomes (24), but not mitral regurgitation.

### 4.2 Interpretation of findings and comparison with existing literature

The pooled reductions are modest in absolute terms but compare favourably with the only established pharmacological benchmark. In the PRIME trial, sacubitril/valsartan lowered regurgitant volume by 7.3 mL (95% CI −12.6 to −1.9) and effective regurgitant orifice area by approximately 30% relative to valsartan over 12 months (25). The effect observed here is of similar or greater magnitude, and was obtained in cohorts already receiving renin–angiotensin system inhibition, beta-blockade and mineralocorticoid receptor antagonism. Its potential relevance is supported by observational data: an effective regurgitant orifice area exceeding 0.2 cm^2^ independently predicts death and heart failure hospitalization (4), and patients whose secondary mitral regurgitation improves with therapy show better long-term survival than non-responders (26). The upper confidence limit (−0.022 cm^2^) nonetheless corresponds to a trivial difference, and the trial-level estimates spanned a four-fold range.

Any mechanism is likely to operate on the ventricle rather than the valve, but the trials point away from the most obvious explanation. Because the regurgitation arises from ventricular dilatation, papillary muscle displacement and annular dilatation, a fall in left ventricular volume would be the expected route to improvement, and SGLT2 inhibitors reduce left ventricular volumes and mass in dedicated remodeling trials (27, 28). Yet neither EFFORT nor DEFORM found a significant between-group difference in left ventricular volumes despite significant reductions in regurgitation (10, 11); only Abou Warda et al., over six months in a predominantly reduced-ejection-fraction cohort, reported concurrent falls in indexed left ventricular mass and volumes (21). Both instead reported improvement in determinants of leaflet closing force and annular geometry: global longitudinal strain and left atrial volume index in EFFORT, and ejection fraction, E/e′, and left atrial volume index in DEFORM. These findings raise the possibility that short-term reductions in regurgitation relate more closely to improved contractile performance, filling pressures, and atrial geometry than to measurable ventricular volume reduction (29). This hypothesis is biologically supported by recent meta-analytic evidence demonstrating that SGLT2 inhibitors significantly attenuate post-ablation atrial fibrillation recurrence and promote left atrial reverse remodeling in heart failure populations (30, 31). Because atrial fibrillation was present in over a quarter of our pooled cohort, left atrial volume unloading and mitigation of myocardial stretch may represent a critical therapeutic pathway. By reducing atrial wall tension and halting progressive annular dilatation, which are mechanisms central to both arrhythmia suppression and atrial functional mitral regurgitation, SGLT2 inhibitors may exert early beneficial geometric effects prior to demonstrable ventricular volumetric regression. No trial was designed to test mediation, however, and improvement in these indices does not establish that they caused the reduction in regurgitation.

Two features of the data temper this account. First, preload reduction can lower measured regurgitation through load-dependent changes in ventricular geometry and transmitral gradient without durable structural change, and DEFORM's three-month follow-up cannot separate a haemodynamic from a remodeling effect; background furosemide was also reduced or discontinued significantly more often in the dapagliflozin arm by three months (*P* = 0.003) (11), so the groups were not volume-matched at follow-up imaging. Second, the heterogeneity observed (I^2^ 72.5% and 63.5%) is better explained clinically than statistically. Baseline severity differed markedly: mean effective regurgitant orifice area was 0.20 cm^2^ in EFFORT, which excluded patients with a planned mitral intervention and in which only 7.8% had an area ≥0.40 cm^2^ (10), against 0.43 to 0.45 cm^2^ in Abou Warda et al. (21). A consistent signal was reported within EFFORT, in which regurgitation severity was the only baseline characteristic to modify the treatment effect: the mean difference was −0.058 cm^2^ below a baseline area of 0.30 cm^2^ and −0.160 cm^2^ at or above it (*P* for interaction = 0.026) (10), the latter close to the −0.180 cm^2^ reported by Abou Warda et al. The observed heterogeneity may therefore partly reflect differences in baseline severity. This remains a hypothesis rather than a finding: trial-level comparisons are susceptible to ecological bias, subgroup interactions derived from small trials are unstable, and confirmation would require adequately powered individual-participant analyses.

The fragility of the estimate compounds this. In leave-one-out analysis, removal of the sole placebo-controlled trial abolished significance for both primary outcomes. The summary effect therefore depends on one trial for its precision while drawing much of its magnitude from the trial at greatest risk of bias: an open-label study in which the allocation sequence was generated by simple randomization without a described concealment mechanism, 7.1% of participants were lost unequally between arms, and mitral regurgitation was a secondary outcome in a trial powered for left ventricular mass (21).

The clinical outcomes warrant comparable caution. Pooled analyses of the major SGLT2 inhibitor outcome trials, comprising more than 20,000 randomized patients, have yielded hazard ratios of approximately 0.72 for first heart failure hospitalization and 0.87 for cardiovascular death (24). The risk ratios of 0.45 and 0.26 reported here are considerably larger, although the two sets of estimates are not directly comparable. A near four-fold greater relative reduction in cardiovascular mortality, derived from 26 events, is unlikely to represent a genuine amplification of benefit in functional mitral regurgitation. Small-study effects, event sparsity, unblinded ascertainment of secondary unadjudicated outcomes and the dominant analytic weight of a single study at some concerns for missing data and selective reporting offer a more parsimonious explanation. The EFFORT and DEFORM investigators were explicit on this point, describing their trials as mechanistic rather than outcome studies and noting that the number of events was insufficient to assess clinical benefit (10, 11).

The divergent results of MITRA-FR, COAPT and RESHAPE-HF2 underline how sensitive the benefit of intervention is to patient selection and to the completeness of medical optimization (32, 33), and contemporary guidance requires medical and device therapy to be optimized before transcatheter edge-to-edge repair is considered (1). SGLT2 inhibition now forms part of that optimization. Current evidence does not, however, show that it reduces the eventual need for intervention, nor that reduction in effective regurgitant orifice area mediates clinical benefit independently of the ventricular actions of these agents.

### 4.3 Limitations

These findings should be interpreted in the context of several limitations. The evidence base comprises only three trials and 455 participants. Although echocardiographic measurements were made blinded to allocation in all three trials, two used an open-label design without a placebo comparator, and background diuretic therapy diverged between arms in one of them, so loading conditions at outcome imaging were not comparable. Generalisability is narrow: the trials were conducted in South Korea, China and Egypt and enrolled predominantly male participants; none included preserved ejection fraction and EFFORT excluded an ejection fraction below 35%, so neither advanced systolic dysfunction nor atrial functional mitral regurgitation is adequately represented. Only dapagliflozin and ertugliflozin were evaluated, with no data for empagliflozin. Reliance on study-level rather than individual participant data precluded subgroup analysis by baseline severity, ejection fraction or atrial fibrillation status, and means that the severity-dependence discussed above could not be tested directly. Another notable limitation of this study is the significant clinical heterogeneity in baseline functional mitral regurgitation severity across the included trials, as seen in the mean baseline effective regurgitant orifice area was 0.20cm^2^ in the EFFORT trial, compared to 0.43cm^2^ in the trial by Abu Warda et al, this nearly two-fold difference in baseline severity likely contributed directly to the substantial statistical heterogeneity observed in our primary outcome analyses. Due to the small number of eligible trials, this heterogeneity between studies was not quantitatively explored and confirmed by a univariate analysis. Finally, adverse events were reported too inconsistently to permit synthesis of safety outcomes.

## 5 Conclusion

In patients with heart failure and chronic functional mitral regurgitation receiving guideline-directed medical therapy, adding an SGLT2 inhibitor was associated with modest reductions in effective regurgitant orifice area and regurgitant volume, together with fewer heart failure hospitalizations and cardiovascular deaths. In two of the three trials these changes occurred without measurable reduction in left ventricular volume. The certainty of the evidence is low for the valvular outcomes and very low for cardiovascular mortality, and the results are hypothesis-generating rather than confirmatory; they do not establish a valve-specific benefit of SGLT2 inhibition. Adequately powered, placebo-controlled randomized trials with standardized loading conditions at echocardiography, adjudicated clinical endpoints and prespecified valvular outcomes are required.

## Data Availability

The datasets presented in this study can be found in online repositories. The names of the repository/repositories and accession number(s) can be found in the article/Supplementary Material.
