# Supplementary material for: Efficacy of sodium-glucose cotransporter-2 inhibitors on functional mitral regurgitation associated with heart failure: a systematic review and meta-analysis of randomized controlled trials
Source: Front Cardiovasc Med. 2026 Oct 21;13:1976876. doi: 10.3389/fcvm.2026.1976876 (PMC13731203; doi:10.3389/fcvm.2026.1976876)
Supplement: Supplementary file 1 [file Table1.docx]

**Supplementary Appendix**

*Efficacy and Safety of Sodium-Glucose Cotransporter-2 (SGLT2) Inhibitors on Functional Mitral Regurgitation Associated with Heart Failure: A Systematic Review and Meta-Analysis of Randomized Controlled Trials*

**Table S1**. Search strategies with results and applied limitations

**Supplementary Table S1**. Search strategies with results and applied limitations

| **Databases** | **Search strategies** | **Results limitations** | **Date of search** | **No. of Results** |
| --- | --- | --- | --- | --- |
| PubMed | (((((((((("heart failure"[MeSH Terms]) OR ("heart failure"[Title/Abstract])) OR ("cardiac failure"[Title/Abstract])) OR ("congestive heart failure"[Title/Abstract])) OR ("hfref"[Title/Abstract])) OR ("hfmref"[Title/Abstract])) OR ("reduced ejection fraction"[Title/Abstract])) OR ("preserved ejection fraction"[Title/Abstract])) OR ("left ventricular dysfunction"[Title/Abstract])) AND ((((((((("sodium glucose transporter 2 inhibitors"[MeSH Terms]) OR ("sglt2 inhibitors"[Title/Abstract])) OR ("sglt2"[Title/Abstract])) OR ("sglt2i"[Title/Abstract])) OR ("sodium glucose cotransporter 2 inhibitor"[Title/Abstract])) OR ("gliflozin"[Title/Abstract])) OR ("dapagliflozin"[Title/Abstract])) OR ("empagliflozin"[Title/Abstract])) OR ("canagliflozin"[Title/Abstract]))) AND (((((("mitral valve insufficiency"[MeSH Terms]) OR ("ventricular remodeling"[Title/Abstract])) OR ("mitral regurgitation"[Title/Abstract])) OR ("mitral insufficiency"[Title/Abstract])) OR ("functional mitral regurgitation"[Title/Abstract])) OR ("cardiac remodeling"[Title/Abstract])) | No limitations applied | 21 July 2026 | 211 |
| Embase | (((((((((('heart failure'/exp) OR ('heart failure':ti,ab,kw)) OR ('cardiac failure':ti,ab,kw)) OR ('congestive heart failure':ti,ab,kw)) OR ('hfref':ti,ab,kw)) OR ('hfmref':ti,ab,kw)) OR ('reduced ejection fraction':ti,ab,kw)) OR ('preserved ejection fraction':ti,ab,kw)) OR ('left ventricular dysfunction':ti,ab,kw)) AND ((((((((('sodium glucose cotransporter 2 inhibitor'/exp) OR ('sglt2 inhibitors':ti,ab,kw)) OR ('sglt2':ti,ab,kw)) OR ('sglt2i':ti,ab,kw)) OR ('sodium glucose cotransporter 2 inhibitor':ti,ab,kw)) OR ('gliflozin':ti,ab,kw)) OR ('dapagliflozin':ti,ab,kw)) OR ('empagliflozin':ti,ab,kw)) OR ('canagliflozin':ti,ab,kw))) AND (((((('mitral valve regurgitation'/exp) OR ('ventricular remodeling':ti,ab,kw)) OR ('mitral regurgitation':ti,ab,kw)) OR ('mitral insufficiency':ti,ab,kw)) OR ('functional mitral regurgitation':ti,ab,kw)) OR ('cardiac remodeling':ti,ab,kw)) | No limitations applied | 21 July 2026 | 69 |
| Cochrane | ([mh "heart failure"] OR "heart failure":ti,ab,kw OR "cardiac failure":ti,ab,kw OR "congestive heart failure":ti,ab,kw OR "reduced ejection fraction":ti,ab,kw AND ([mh "sodium glucose transporter 2 inhibitors"] OR "sglt2 inhibitors":ti,ab,kw OR "sglt2i":ti,ab,kw OR "dapgaflozin":ti,ab,kw OR "empagliflozin":ti,ab,kw OR "gliflozin":ti,ab,kw) AND ([mh "mitral valve insufficiency"] OR "ventricular remodeling":ti,ab,kw OR "mitral regurgitation":ti,ab,kw OR "mitral insufficiency":ti,ab,kw OR "functional mitral regurgitation":ti,ab,kw OR "cardiac remodeling":ti,ab,kw) | No limitations applied | 21 July 2026 | 72 |
| Scopus | TITLE-ABS-KEY ( "heart failure" ) OR ( "cardiac failure" ) OR ( "congestive heart failure" ) OR ( "hfref" ) OR ( "reduced ejection fraction" ) OR ( "left ventricular dysfunction" ) AND TITLE-ABS-KEY ( "sodium glucose transporter 2 inhibitors " ) OR ( "sglt2 inhibitors " ) OR ( "sglt2i" ) OR ( "dapagliflozin" ) OR ( "empagliflozin" ) OR ( "canagliflozin" ) AND TITLE-ABS-KEY ( "mitral valve insufficiency" ) OR ( "ventricular remodeling" ) OR ( "mitral regurgitation" ) OR ( "mitral insufficiency" ) OR ( "functional mitral regurgitation" ) | No limitations applied | 21 July 2026 | 286 |
| Web of Science | TS=("heart failure" OR "cardiac failure" OR "congestive heart failure" OR "hfref" OR "reduced ejection fraction" OR "left ventricular dysfunction") AND TS=("sodium glucose transporter 2 inhibitors" OR "sglt2 inhibitors" OR "sglt2i" OR "dapagliflozin" OR "empagliflozin" OR "canagliflozin") AND TS=("mitral valve insufficiency" OR "ventricular remodeling" OR "mitral regurgitation" OR "mitral insufficiency" OR "functional mitral regurgitation") | No limitations applied | 21 July 2026 | 107 |
